# Development and validation of a comprehensive machine learning framework for a diagnostic model of uremia based on genes involved in major depressive disorder

**DOI:** 10.3389/fneph.2025.1576349

**Published:** 2025-10-02

**Authors:** Kaiyao Jiang, Chi Zhang, Cheng Shen, Xingxing Fang, Huaxing Huang, Bing Zheng

**Affiliations:** ^1^ Department of Urology, Affiliated Hospital 2 of Nantong University, Nantong, Jiangsu, China; ^2^ Jiangsu Nantong Urological Clinical Medical Center, Nantong, Jiangsu, China; ^3^ Department of Emergency Medicine, the Afffliated Suqian Hospital of Xuzhou Medical University, Suqian, China; ^4^ Department of Nephrology, Sir Run Run Hospital, Nanjing Medical University, Nanjing, China; ^5^ Department of Nephrology, Affiliated Hospital 2 of Nantong University, Nantong, Jiangsu, China

**Keywords:** uremia, major depressive disorder, machine learning, diagnostic models, bioinformatics

## Abstract

**Background:**

Major depressive disorder (MDD) and uremia are two chronic wasting diseases that have interactive effects and significantly aggravate patients’ distress. However, the molecular basis linking these diseases remains poorly investigated.

**Methods:**

Various machine learning algorithms were used to analyze transcriptome data from the Gene Expression Omnibus (GEO) datasets, including those from MDD and uremia patients, to develop and validate our model. After removing batch effects, differentially expressed genes (DEGs) were identified between each disease group and the control group. Functional enrichment analysis was then performed at the intersection of DEGs from the two diseases. In addition, single-sample gene set enrichment analysis (ssGSEA) quantitative immune infiltration analysis was conducted. The optimal diagnostic model of uremia was constructed by analyzing and verifying the training set with multiple combinations of 12 machine learning algorithms. Finally, potential drugs for uremia were identified using the “Enrichr” platform.

**Results:**

According to enrichment analysis, a total of seven key genes closely related to MDD and uremia, mainly involved in the immune process, were identified. Immune infiltration analysis showed that MDD and uremia had different profiles of immune cell infiltration compared to healthy controls. Powerful diagnostic markers of seven genes (IL7R, CD3D, RETN, RAB13, TNNT1, HP, and S100A12) were constructed from these genes, and all showed better performance than published uremia diagnostic models. In addition, decitabine and nine other agents were found to be potential agents for the treatment of uremia.

**Conclusion:**

Our study combined bioinformatics techniques and machine learning methods to develop a diagnostic model for uremia, focusing on common genes between MDD and uremia.

## 1 Introduction

Major depressive disorder (MDD) is a prevalent psychiatric disorder with a significant global impact, causing substantial disability and affecting everyday functioning (1). Its clinical symptoms include persistent depressed mood, anhedonia, fatigue, feelings of worthlessness, and impaired cognitive performance (2). Major depression is estimated to have a lifetime prevalence of up to 19% (3), placing a significant burden on society (4). It remains a challenge in the treatment of as many as half of the cases (5). Based on previous studies, uremia has a significant association with MDD. For example, studies conducted by Heng-Jung Hsu et al. showed that the incidence of depressive disorders was significantly higher in uremia patients (6). Depression can have a serious impact on people’s lives, even letting people give up life, so it is urgent to explore the association between uremia and depression.

Uremia is the final stage of chronic renal failure. It is clinically characterized by abnormal water, electrolyte, acid, and base balance and increased levels of metabolites (e.g., creatinine and urea) in the blood (7). The uremic phase is often associated with some secondary conditions and complications of chronic kidney disease (CKD), including renal function, circulatory system, endocrine, and metabolic disorders, as well as neuromuscular dysfunction and cognitive impairment (8, 9). Among them, MDD is a more common complication of uremia. Uremia is a chronic wasting disease that usually requires hemodialysis treatment, and since the introduction of dialysis, the mental health of hemodialysis patients has been the focus of research (10, 11). Kimmel et al. (12) demonstrated that persistent depression is a risk factor for death in hemodialysis patients. Therefore, it is crucial to construct a diagnostic model of uremia associated with major depression to control it in time at an early stage and reduce mortality. However, the diagnosis of Uremia mainly depends on serum creatinine and glomerular filtration rate, which makes the diagnosis of Uremia very lacking. In addition, although many genetic markers have been investigated, such as CNOT8, MST4, PPP2CB, PCSK7, and RBBP4, none of them could demonstrate enough specificity and sensitivity for clinical applications (13). Others have shown a bidirectional relationship between depression and physical diseases such as chronic kidney disease (14). Therefore, it is particularly important to construct a better diagnostic model that can be applied in clinical practice through the relationship between depression and chronic kidney disease for the early detection of uremia.

Bioinformatics and machine learning techniques have evolved significantly over the last decade, and this is how we can investigate potential biomarkers and therapeutics for diseases (15–18). In this study, we used multiple integrated bioinformatics tools to reveal hub genes and underlying mechanisms linking uremia and MDD by analyzing data from three uremia datasets and three MDD datasets selected from the Gene Expression Omnibus (GEO) database. We explored immune cell infiltration in uremia and MDD. In addition, 113 combined machine learning algorithm frameworks were used to construct a uremia diagnostic model.

## 2 Methods

### 2.1 Data collection

Appropriate datasets were filtered from the GEO database. First, datasets of transcriptomes for major depression and uremia or end-stage renal disease were searched. Then, because multiple datasets were included, the data in the dataset were kept as much as possible above 6. Finally, it was ensured that the included dataset was suitable for machine learning methods. Following the above steps, the following six datasets were obtained from the National Center for Biotechnology Information (NCBI) GEO (https://www.ncbi.nlm.nih.gov/geo/): GSE37171, GSE38750, GSE43484, GSE52790, GSE76826, and GSE98793 (9, 19–23). These datasets are described in detail in **Table 1** and include the microarray platform, panel, and number of samples.

**Table 1:** Basic information of GEO datasets used in the study.

ID	GSE series	Disease	Samples	Source types	Platform
1	GSE37171	Uremia	63 uremia patients and 20 normal controls	Whole blood	GPL570
2	GSE38750	Uremia	15 uremia patients and 19 normal controls	Iliac artery and renal artery	GPL571
3	GSE43484	Uremia	3 uremia patients and 3 normal controls	Monocyte	GPL571
4	GSE52790	MDD	10 MDD patients and 12 normal controls	Peripheral blood	GPL17976
5	GSE76826	MDD	20 MDD patients and 12 normal controls	Blood	GPL17077
6	GSE98793	MDD	128 MDD patients and 64 normal controls	Whole blood	GPL570

GEO, Gene Expression Omnibus; MDD, major depressive disorder.

### 2.2 Removal of batch effect

Before performing analysis, we merged the three MDD datasets mentioned in **Table 1** (GSE52790, GSE76826, and GSE98793). We then corrected batch effects using the “ComBat” function in the “sva” package (version 3.52.0) (24). We used principal component analysis (PCA) analysis to assess the validity of this correction. Using the same method, we then corrected three uremia cohorts (GSE37171, GSE38750, and GSE43484).

### 2.3 Determination of DEGs

In the analysis of the MDD and uremia datasets, the “Limma” package (25) within the R software was employed to identify differentially expressed genes (DEGs). Our selection criteria, requiring |log_2_FC| > 0.25 and p-value <0.05, ensured a comprehensive and accurate analysis. The outcomes were visually represented through compelling volcano plots, and the shared part of the two sets of DEGs was effectively depicted using Venn diagrams. To further investigate the shared genes, Protein-Protein Interaction Networks (PPI) networks were confidently generated using GeneMANIA, facilitating an insightful exploration of their associations (http://genemania.org/).

### 2.4 Enrichment analysis of common genes in uremia with MDD

To gain insights into the biological functions and mechanistic pathways of common genes, we utilized the “org.Hs.eg.db”, “ggplot2”, “clusterProfiler”, “enrichplot”, “GSEABase”, and “DOSE” packages to conduct Gene Ontology (GO) and Kyoto Encyclopedia of Genes and Genomes (KEGG) pathway enrichment analyses, as well as Disease Ontology Semantic and Enrichment (DOSE). p < 0.05 for enrichment assessment was considered significant.

### 2.5 Immune cell infiltration

The quantification of 23 infiltrating immune cells in both diseases was conducted using single-sample gene set enrichment analysis (ssGSEA). Then, the differential expression of immune cells in normal and uremia patients was further studied and analyzed.

### 2.6 Machine learning algorithms

Twelve machine learning algorithms were used to construct 113 different models: Lasso, Ridge, Stepglm, XGBoost, Random Forest (RF), Elastic Net (Enet), Partial Least Squares Regression for Generalized Linear Models (plsRglm), Generalized Boosted Regression Modeling (GBM), NaiveBayes, Linear Discriminant Analysis (LDA), Generalized Linear Boosting (glmBoost), and Support Vector Machine (SVM). First, the raw data were preprocessed to eliminate the influence of different feature scales. Then, the dataset was randomly divided into training and testing sets, 70% of which were training sets and 30% of which were testing sets. During the model training phase, a variety of machine learning algorithms were used to evaluate their performance. These models were trained on the training set, and the hyperparameters were optimized using cross-validation. During the model evaluation phase, Area Under Curve (AUC) values were calculated for each model using the test set (threshold set at 0.7) to measure their classification performance. Finally, AUC values were calculated for each model using the RunEval function, and heat maps were generated using the SimpleHeatmap function to visualize the performance of each model. The model with the highest AUC value was selected as the best model (26). In addition, calibration curves were used to assess the predictive performance of our diagnostic model, Decision Curve Analysis (DCA) curves were generated to assess the clinical utility of the model, and Nomo plots were also generated to calculate the probability of disease occurrences. Finally, the DeLong test was used to compare our model’s diagnostic performance with that of two existing uremia diagnostic models (13, 27).

### 2.7 Candidate drug identification

To explore drugs that may target the mechanisms of action in uremia and MDD, we utilized the Drug Signatures Database (DSigDB) within the Enrichr web platform (https://maayanlab.cloud/Enrichr/).

### 2.8 Statistical analysis

Statistical analyses were performed using the R software version 4.4.1. An unpaired Student’s t-test compared differences between the two groups. *p* < 0.05 was considered statistically significant.

## 3 Results

### 3.1 Data processing

**Figure 1:**
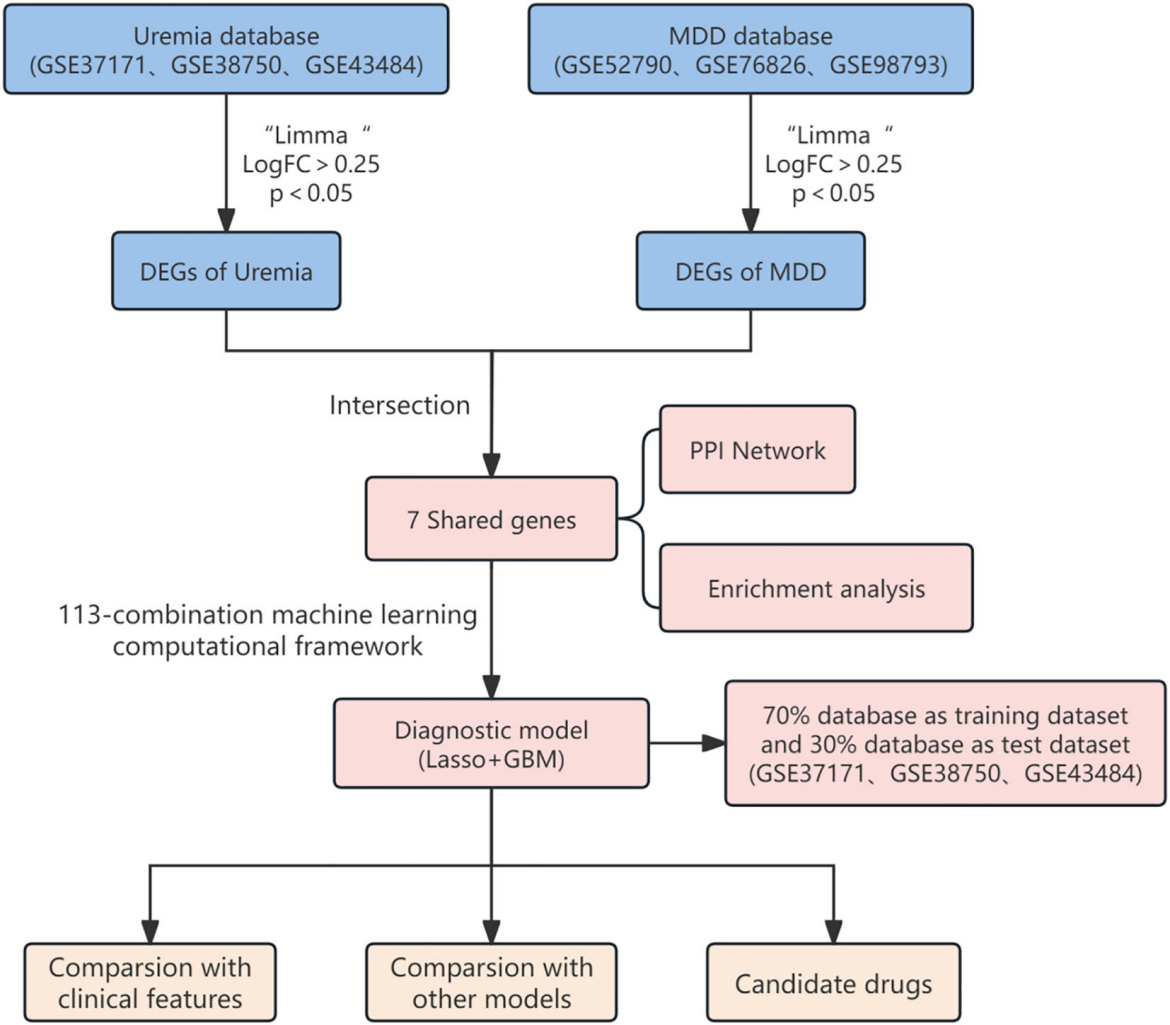
The flowchart of the study.

**Figure 2:**
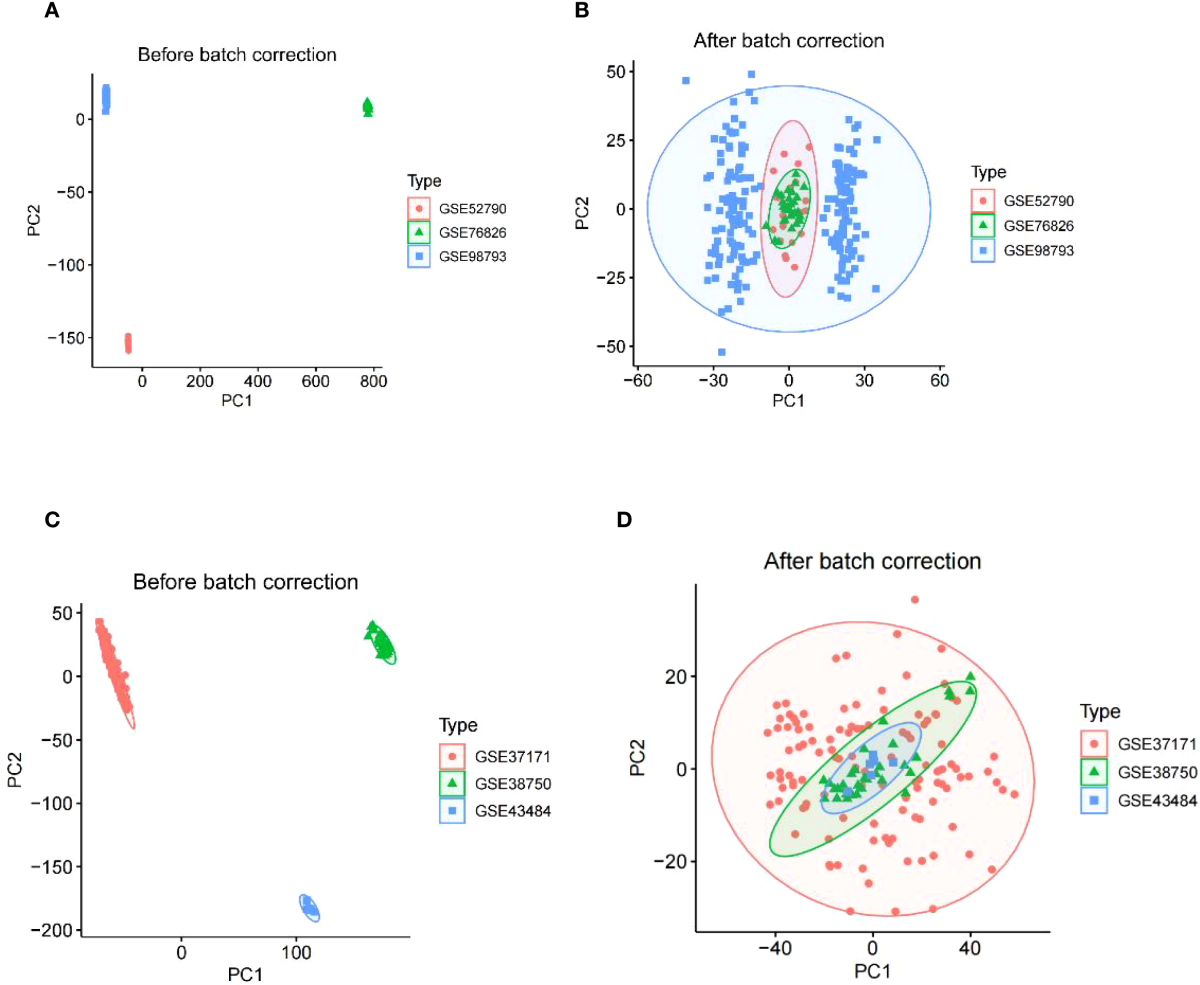
The integration of MDD datasets and uremia datasets. **(A)** PCA of three raw MDD datasets without batch effect correction. **(B)** PCA of the integrated MDD dataset after batch effect correction. **(C)** PCA of three original uremia datasets before batch effect correction. **(D)** PCA for the combined uremia dataset after batch effect correction. MDD, major depressive disorder.

The study design flowchart is shown in **Figure 1**. Original MDD and control transcriptome data were obtained from GEO, integrated after removing batch effects, and standardized MDD case and control processing cohorts were generated (**Figures 2A, B**). Similarly, the post-batch corrections of the original uremia and control cohorts were combined (**Figures 2C, D**) to obtain a standardized validation cohort with markedly reduced batch effects.

### 3.2 Identification of differential expression associated with uremia and MDD

**Figure 3:**
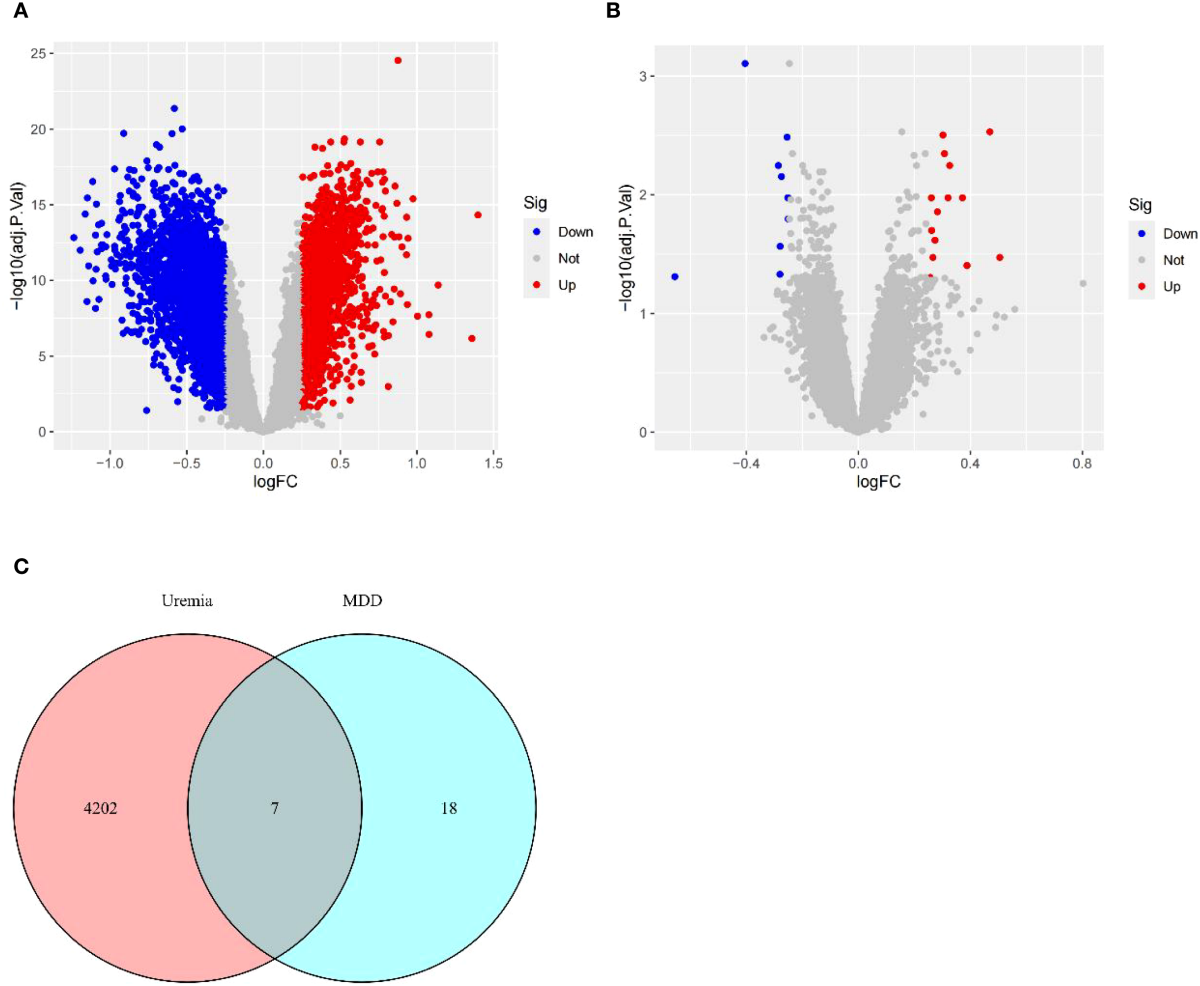
Identification of DEGs. **(A)** Volcano plots describing DEGs between uremia and healthy controls. **(B)** Volcano plots showing DEGs between MDD and healthy controls. **(C)** Venn diagram revealing seven DEGs shared between uremia and MDD. DEGs, differentially expressed genes; MDD, major depressive disorder.

Based on the relationship between MDD and uremia, limma analysis was performed for the uremia (GSE37171, GSE38750, and GSE43484) and MDD (GSE52790, GSE76826, and GSE98793) cohorts to identify causative genes for MDD-associated uremia. Among the 4,209 DEGs detected in the uremia cohort, 1,871 genes showed upregulated expression, while 2,338 were downregulated (**Figure 3A**). The MDD cohort yielded 25 DEGs, 15 of which were upregulated and 10 downregulated (**Figure 3B**). The DEGs of uremia and MDD were intersected to obtain seven shared genes for constructing a diagnostic model of uremia (**Figure 3C**).

### 3.3 Functional enrichment of the shared genes

**Figure 4:**
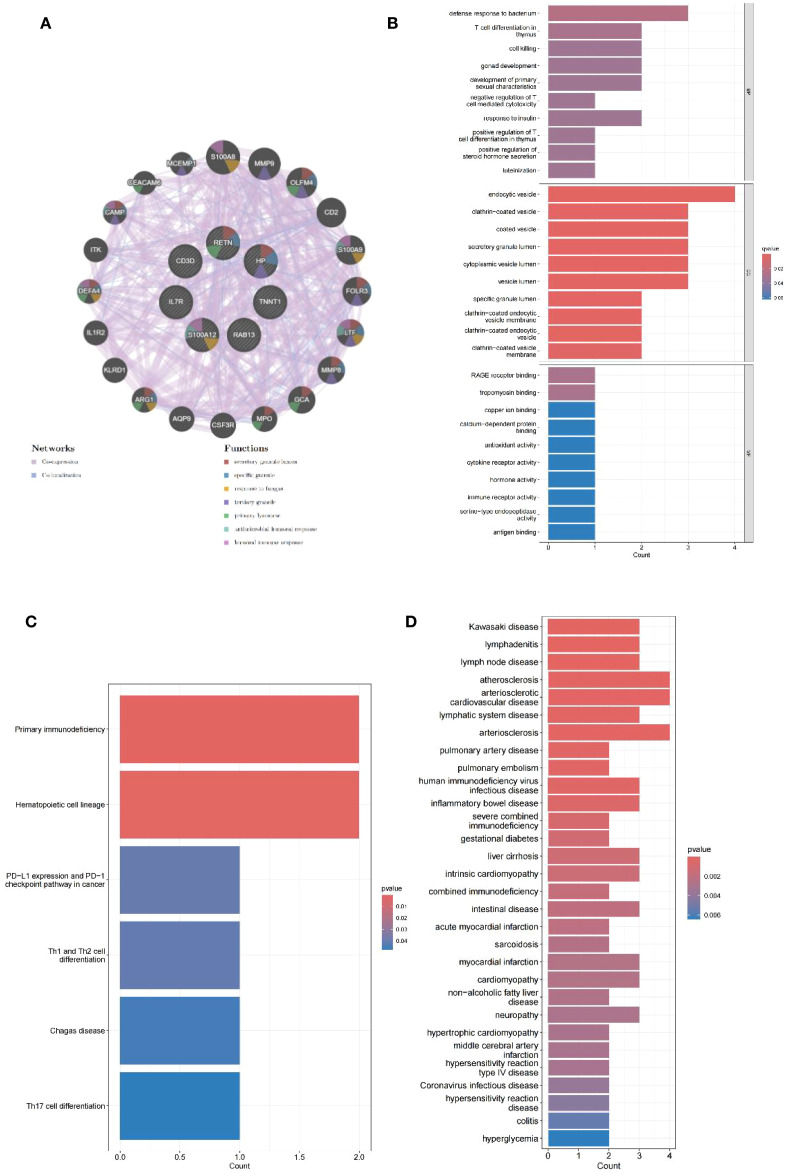
PPI network analysis and enrichment analysis. **(A)** PPI network of seven shared genes constructed using GeneMANIA. **(B)** Bar plots of GO enrichment analysis results for biological process, cellular component, and molecular function. **(C)** Bar plots of KEGG pathway enrichment analysis. **(D)** Bar plots of Disease Ontology enrichment analysis.

The PPI networks of the shared genes were established from the GeneMANIA database (**Figure 4A**), and then GO, KEGG, and DOSE were used for functional enrichment analysis and to search for potential pathogenic mechanisms. GO enrichment analysis showed overexpression of biological processes, including defense response to bacterium, T-cell differentiation in the thymus, cell killing, gonad development, development of primary sexual characteristics, negative regulation of T cell-mediated cytotoxicity, response to insulin, positive regulation of T-cell differentiation in the thymus, positive regulation of steroid hormone secretion, and luteinization. Enriched cellular components included endocytic vesicle, clathrin-coated vesicle, coated vesicle, secretory granule lumen, cytoplasmic vesicle lumen, vesicle lumen, specific granule lumen, clathrin-coated endocytic vesicle membrane, clathrin-coated endocytic vesicle, and clathrin-coated vesicle membrane. Overexpressed molecular functions included receptor for advanced glycation end products (RAGE) receptor binding, tropomyosin binding, copper ion binding, calcium-dependent protein binding, antioxidant activity, cytokine receptor activity, hormone activity, immune receptor activity, serine-type endopeptidase activity, and antigen binding (**Figure 4B**). KEGG pathway analysis further revealed primary immunodeficiency, hematopoietic cell lineage, PD-L1 expression and PD-1 checkpoint pathway in cancer, Th1 and Th2 cell differentiation, Chagas disease, and T-helper 17 (Th17) cell differentiation (**Figure 4C**). Disease Ontology Semantic and Enrichment analysis also showed Kawasaki disease, lymphadenitis, lymph node disease, atherosclerosis, arteriosclerotic cardiovascular disease, lymphatic system disease, arteriosclerosis, pulmonary artery disease, pulmonary embolism, human immunodeficiency virus infectious disease, inflammatory bowel disease, severe combined immunodeficiency, gestational diabetes, liver cirrhosis, intrinsic cardiomyopathy, combined immunodeficiency, intestinal disease, acute myocardial infarction, sarcoidosis, myocardial infarction, cardiomyopathy, non-alcoholic fatty liver disease, neuropathy, hypertrophic cardiomyopathy, middle cerebral artery infarction, hypersensitivity reaction type IV disease, coronavirus infectious disease, hypersensitivity reaction disease, colitis, and hyperglycemia (**Figure 4D**).

### 3.4 Analysis of immune cell infiltration in uremia and MDD

**Figure 5:**
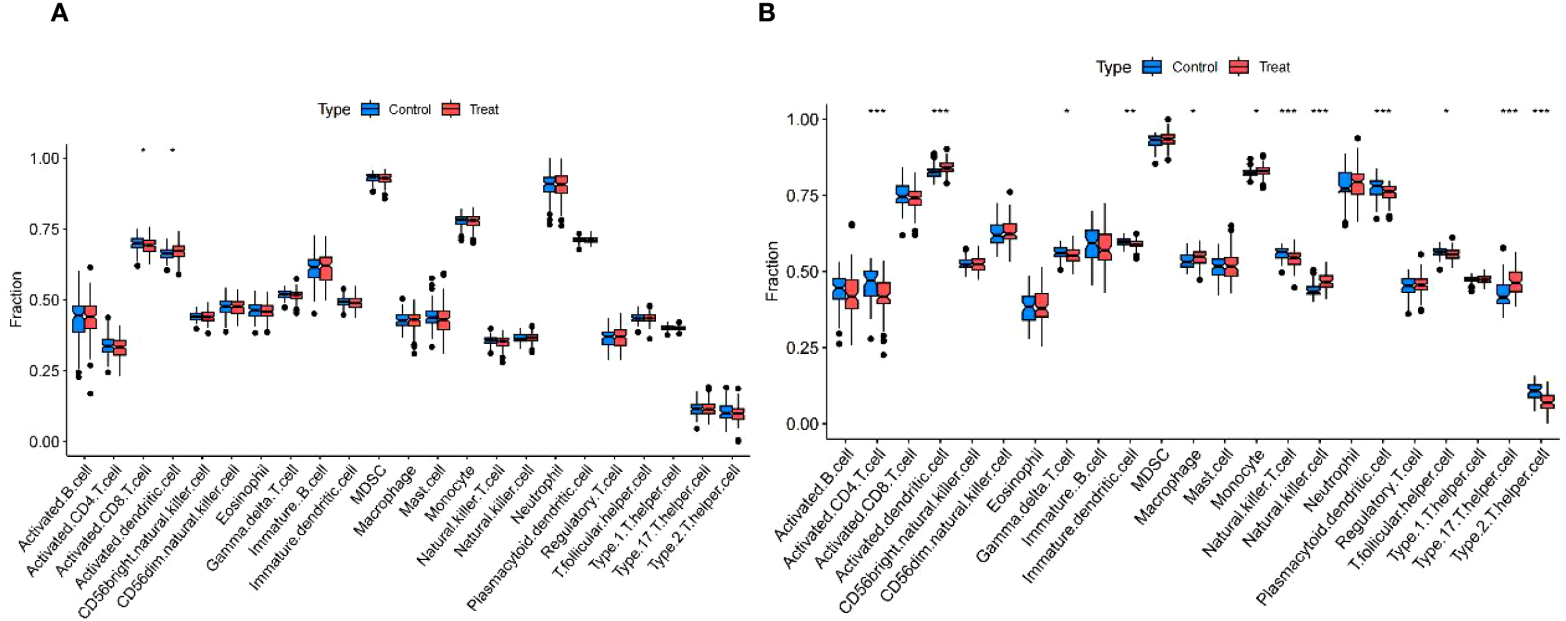
Immunological features of MDD and uremia. **(A)** Boxplots comparing immune cell abundances between MDD and controls. **(B)** Boxplots comparing immune cell abundances between uremia and controls. *** p < 0.001,** p < 0.01, and *p < 0.05. MDD, major depressive disorder.

The enrichment analysis of the shared genes between uremia and MDD showed a significant association with immune cell infiltration and the development of inflammation. ssGSEA was used to describe the composition of immune cell subsets in the uremia and MDD cohorts. MDD samples exhibited decreased activated CD8 T cells and increased activated dendritic cells relative to control samples (**Figure 5A**). The box plot (**Figure 5B**) indicates that compared to controls, in the uremia cohort, activated dendritic cells, macrophages, monocytes, natural killer cells, and type 17 T-helper cell proportion increased, while activated CD4 T cells, immature dendritic cells, natural killer T cells, plasmacytoid dendritic cells, T follicular helper cells, type 2 T-helper cells, and gamma delta T-cell abundance decreased.

### 3.5 Identification of diagnostic hub genes by machine learning and establishment of a diagnostic model for MDD-associated uremia

**Figure 6:**
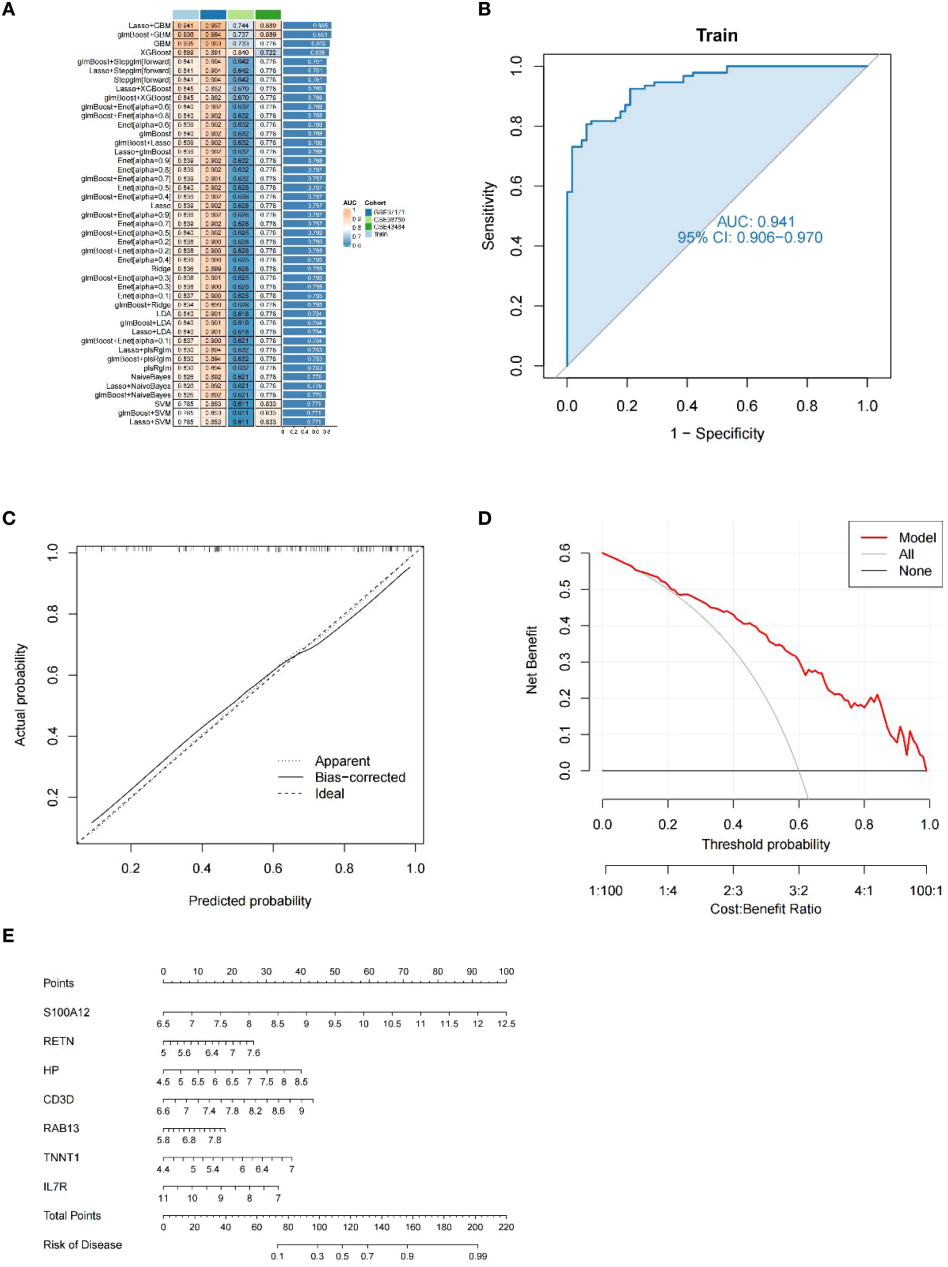
Diagnostic performance of our model. **(A)** A total of 113 machine learning algorithm combinations evaluated by 10-fold cross-validation. **(B)** ROC curves for the training cohort. **(C)** Calibration curve for the training cohort. **(D)** DCA curves for the training cohort. **(E)** Nomo plot of the training cohort.

The most robust diagnostic model, based on seven shared genes, was constructed by reducing selection bias using 113 combinations of 12 machine learning algorithms. The analysis was performed on a training dataset that randomly assigned 70%, and the remaining 30% test set was used to evaluate the predictive performance of diagnostic models (**Figures 6A, B**). By integrating the Lasso and GBM algorithms, the final model that showed the best performance was built. The AUC value of the Receiver Operating Characteristic (ROC) curve was obtained to be 0.941, and the constructed model had superior predictive performance. The Lasso + GBM algorithm identified seven key genes (IL7R, CD3D, RETN, RAB13, TNNT1, HP, and S100A12). The calibration curve of our diagnostic model, such as 6C, the bias-corrected line obtained by bootstrap sampling, was close to the ideal diagonal of the cohort, visually showing that the predicted probability of the model was highly consistent with the actual observed probability, and once again proved the accuracy of the model. A DCA curve analysis was also conducted (**Figure 6D**), the curve shows that from a threshold probability of approximately 0.2, the net gain of intervention according to the prediction model begins to be significantly higher than that of complete intervention or no intervention. Although the net gain decreases with increasing threshold probability values, it is still significantly stronger than that of intervention with full or no, so it can be seen that this model has a practical application value. Finally, as shown in **Figure 6E**, the integration analysis of the seven genes established a Nomo plot, facilitating a more convenient estimation of the probability of having uremia based on patient test results in clinical practice.

### 3.6 Subgroup analysis of uremia diagnostic model

**Figure 7:**
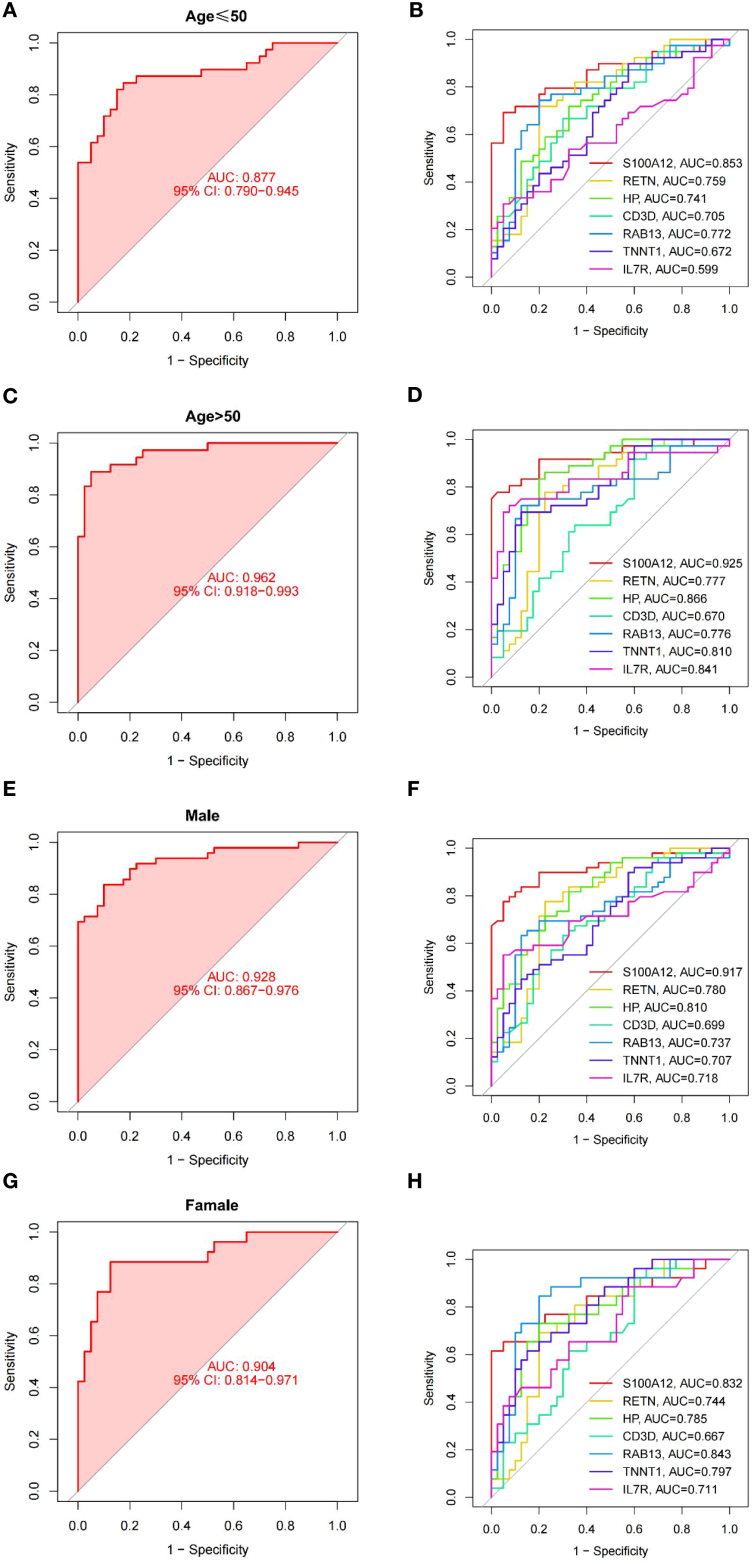
ROC curves for model in subgroups. **(A, B)** ROC curves for the young (age ≤ 50 years) subgroup of model **(A)** and each gene **(B)**. **(C, D)** ROC curves for the old (age > 50 years) subgroup of model **(C)** and each gene **(D)**. **(E, F)** ROC curves for the male subgroup of model **(E)** and each gene **(F)**. **(G, H)** ROC curves for the female subgroup of model **(G)** and each gene **(H)**.

We performed a subgroup analysis of the predictive model that demarcated age by 50 (**Figures 7A–D**). In contrast, the diagnostic performance of the predictive model was higher in the age > 50 group, with AUC values reaching 0.962 (**Figure 7C**), and ROC curve analysis was also performed for each gene. It can be seen that S100A12 has the highest predicted AUC value regardless of age (**Figure 7D**). In addition to age, we also analyzed gender (**Figures 7E–H**). We found that the accuracy of predicting men was higher than that of women, but weaker than that of the overall prediction model (**Figures 7E, G**). Then, we analyzed each gene (**Figures 7F, H**) and found that S100A12 still had the highest AUC value.

### 3.7 Comparison of uremia diagnostic models

**Figure 8:**
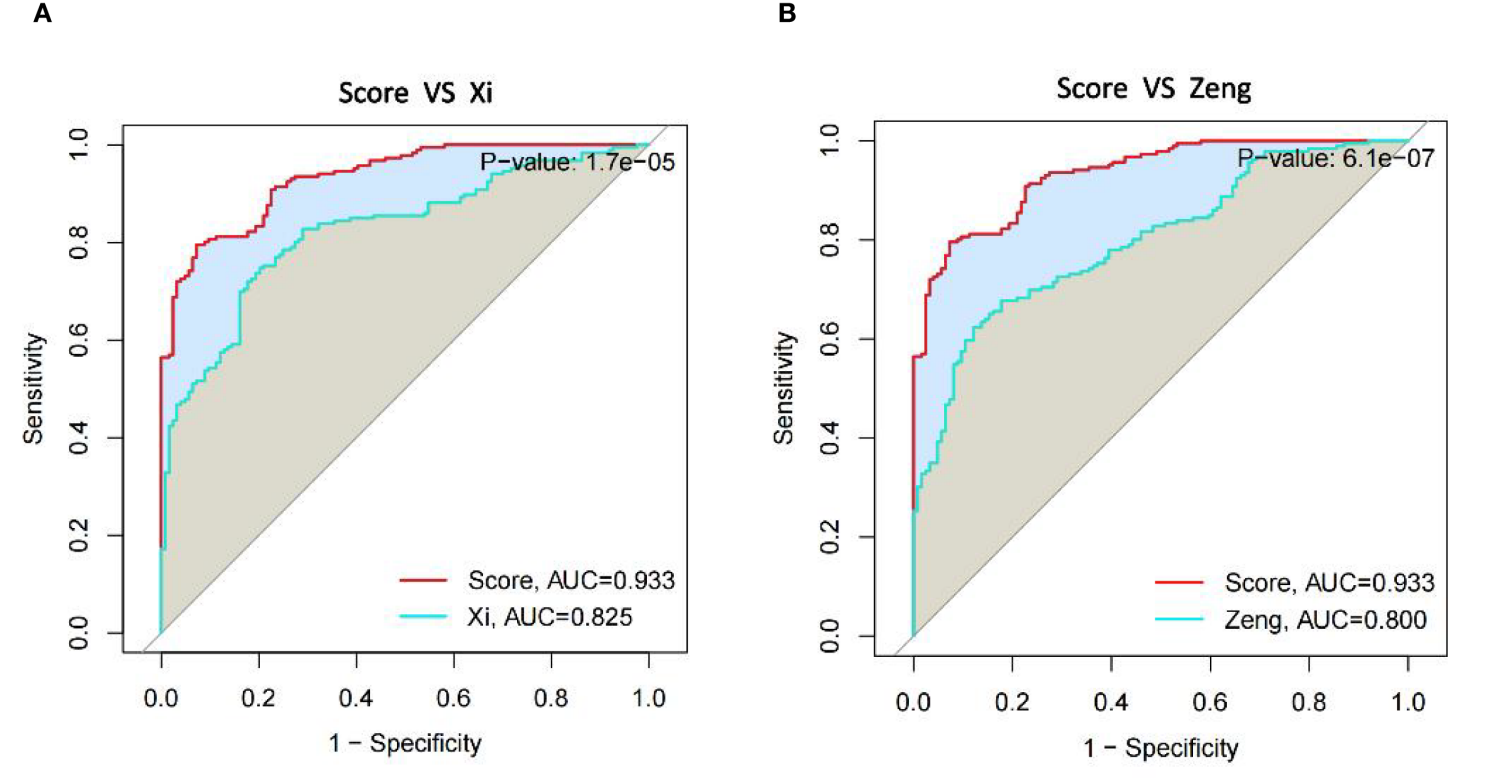
Comparison of diagnostic gene expression features in uremia. **(A)** ROC curves comparing our model to Xi et al. model in the uremia dataset. **(B)** ROC curves comparing our model with the Zeng et al. model in the uremia dataset.

Because of the developments of bioinformatics and big data research technology, many diagnostic models for uremia have recently been developed that combine machine learning methods. Comprehensively comparing the performance of our model with that of other models, it was found that our prediction model performed better than both of them in comparison with the Zeng model (13) of network-based variable selection method (**Figure 8A**) and the Xi model (27) analysis of cellular senescence-associated genes (**Figure 8B**).

### 3.8 Candidate drug identification

Genes in diagnostic models were analyzed using the DSigDB drug database on Enrichr to find potential targeted drugs. The top 10 candidates were decitabine, retinol, atorvastatin, liothyronine, hexachloroethane, cholesterol, simvastatin and niacin, hydrocortisone, dexamethasone, and caspan (**Table 2**).

**Table 2:** Uremia and major depressive disorder (MDD) gene-targeted drugs.

Term	p-Value	Odds ratio	Combined score	Genes
Decitabine	0.01931202	7.594323873	29.97500571	TNNT1; RAB13; IL7R
Retinol	9.29E−04	59.72932331	417.0006812	HP; RETN
Atorvastatin	0.0010413	56.3177305	386.7499407	TNNT1; RETN
Liothyronine	0.001770571	42.82810811	271.3783008	HP; RETN
Hexachloroethane	0.001827075	42.13829787	265.6836032	HP; RETN
Cholesterol	0.001827075	42.13829787	265.6836032	HP; RETN
Simvastatin and niacin	0.001884438	41.47015707	260.1889826	TNNT1; RETN
Hydrocortisone	0.001981945	40.40204082	251.4492303	HP; RETN
Dexamethasone	0.008655043	18.68639618	88.75315333	HP; IL7R
Caspan	0.029904067	9.522084367	33.42023831	TNNT1; RAB13

## 4 Discussion

Both uremia and MDD have a significant impact on the physical and mental health of patients, and extensive research has been conducted on the relationship between these two diseases (28, 29). However, further investigation is necessary to explore the genetic interaction between them.

The emergence of microarray and sequencing technologies has facilitated the exploration of disease processes and molecular landscapes. Furthermore, the increasing development of bioinformatics analysis and machine learning has allowed us to analyze massive datasets, explore meaningful biomarkers, understand the potential mechanisms of action of diseases, and develop promising therapeutic drugs. These advancements offer valuable perspectives on the advancement and novel avenues of complex diseases (30–32). As far as we know, this is the first study to use 12 machine learning algorithms combined with biological information to reveal MDD-associated uremic pathogenic genes. Furthermore, as our diagnostic model predominantly utilizes blood specimens from patients, assessing the levels of diagnostic genes in the blood can help estimate the risk of uremia. This offers a clinically easy-to-perform method for diagnosis. In conclusion, the diagnostic model we explored holds significant promise for achieving early screening of uremia patients and interventions, thereby improving the outcomes of uremia patients.

A total of 4,209 DEGs between uremia and normal, and 25 DEGs between MDD and normal were analyzed using GEO’s dataset. DEGs at the intersection of uremia and MDD were taken to obtain seven common risk genes. PPI networks were constructed using GeneMANIA. GO and KEGG enrichment analyses were then performed, and some biological behaviors and action pathways were found, suggesting a potential mechanism for uremia development and MDD development. GO enrichment analysis highlighted factors such as defense response to bacterium, T-cell differentiation in the thymus, cell killing, gonad development, development of primary sexual characteristics, negative regulation of T cell-mediated cytotoxicity, response to insulin, positive regulation of T-cell differentiation in the thymus, positive regulation of steroid hormone secretion, and luteinization. Enriched cellular components included endocytic vesicle, clathrin-coated vesicle, coated vesicle, secretory granule lumen, cytoplasmic vesicle lumen, vesicle lumen, specific granule lumen, clathrin-coated endocytic vesicle membrane, clathrin-coated endocytic vesicle, and clathrin-coated vesicle membrane. Overexpressed molecular functions included RAGE receptor binding, tropomyosin binding, copper ion binding, calcium-dependent protein binding, antioxidant activity, cytokine receptor activity, hormone activity, immune receptor activity, serine-type endopeptidase activity, and antigen binding. In addition, KEGG pathway analysis showed significant enrichment of pathways associated with primary immunodeficiency, hematopoietic cell lineage, PD-L1 expression and PD-1 checkpoint pathway in cancer, Th1 and Th2 cell differentiation, Chagas disease, and Th17 cell differentiation.

In the KEGG enriched pathway, there is a potential association between Th17 differentiation and the onset of uremia and MDD. Research indicates a significant increase in Th17 cells in the peripheral blood of MDD patients (33). Similarly, uremia patients show a discernible rise in these immune cells when compared to healthy controls, suggesting a possible correlation between uremia and the upregulation of Th17 cells (34). The lack of significant change in their levels following dialysis in observed patients does not exclude the potential for uremia progression linked to immune activation. Further comprehensive studies are necessary to clarify this relationship. Notably, previous research has shown that these cells play a role in advancing atherosclerosis (35, 36). Interleukin-17 (IL-17), produced by Th17 cells, has synergistic effects with tumor necrosis factor-α (TNF-α), which contributes to the pathogenesis of atherosclerotic vascular diseases by creating a pro-inflammatory microenvironment (37). The proliferation of these immune cells could not only contribute to the onset of uremia but also increase susceptibility to cardiovascular complications in affected patients. It is well established that they play a significant role in mediating autoimmunity (38, 39), which suggests that uremia may have some relationship with the primary immunodeficiency pathway. Additionally, Disease Ontology Semantic and Enrichment analysis indicates that uremia may be complicated by Kawasaki disease, lymph node disease, arteriosclerosis disease, pulmonary artery disease, pulmonary embolism, human immunodeficiency virus disease, and inflammatory bowel disease.

The occurrence and development of uremia may be associated with immune activation (40), so we analyzed the immune expression of uremia and found that in patients with uremia, activated dendritic cells, macrophages, monocytes, natural killer cells, and type 17 T-helper cell proportion increased, while activated CD4 T cells, immature dendritic cells, natural killer T cells, plasmacytoid dendritic cells, T follicular helper cells, type 2 T-helper cells, and gamma delta T-cell abundance decreased. Our immune cell analysis is also consistent with previous studies suggesting that several immune cells, including dendritic cells and macrophages, are activated and may contribute to the development of CKD or even uremia (41–43). Our analysis also suggests a decrease in several immune cells, possibly because the immune system of uremia patients is overactivated but functionally compromised. Still, few relevant studies prompt us to investigate the immune cell infiltration and mechanisms of uremia further.

Uremia is now being diagnosed at a more advanced stage, prompting a heightened emphasis on early detection and disease management. The application of machine learning techniques to construct diagnostic models for diseases and predict patient survival has gained significant attention. Nevertheless, successfully translating these methods into clinical practice while ensuring diagnostic and predictive accuracy presents a notable challenge. Certain studies have developed diagnostic models for uremia using specific algorithms and conducted screenings for differential genes. However, it is important to note that these endeavors may be susceptible to personal biases and inherent preferences (26, 44). Thus, we employed 113 combinations of 12 machine learning algorithms to compare their diagnostic performance and identify the best model that mitigates bias caused by these factors, and ultimately, we determined Lasso + GBM as the best model. This study approach significantly reduces the complexity of research and uncovers the most representative patterns, enabling the development of a streamlined and more meaningful model. To further analyze the performance of prediction models constructed using multiple machine learning algorithms, we selected two published uremic diagnostic models that associate with different functional genes. One was Zeng’s model (13), which included two GEO datasets, GSE37171 and GSE70528, and associated modules were identified using the Weighted gene co-expression network analysis (WGCNA) method, followed by Lasso regression, to identify five genes predictive of end-stage renal disease. The other was Xi’s model (27), which incorporated the GEO dataset GSE37171 to screen five predictive genes of end-stage renal disease associated with cellular senescence through a PPI interaction network. As can be seen from the results, our prediction model performs significantly better than the other two models. However, our model has two more genes than the other models, and this increase in the number of genes may bring a little difficulty in clinical practice. Future efforts should, therefore, focus on the simple and efficient analysis of more models, ensuring superior predictive performance and enabling widespread implementation in clinical settings.

It is important to note that in a previous study, haptoglobin (HP) in seven model genes that comprise our diagnostic model is linked to hemolytic uremia (45). Mouse experiments have shown that mice with hemolytic uremic syndrome (HUS) lacking haptoglobin have a 25% reduction in survival compared with normal mice. When low doses of haptoglobin were administered to Shiga toxin-challenged wild-type mice, it reduced renal platelet deposition and neutrophil recruitment, suggesting that haptoglobin has beneficial effects, at least partly. Additionally, S100A12 has been found to be a strong predictor of cardiovascular mortality in end-stage renal disease (46–48). It has been discovered that RAGE triggers pro-inflammatory pathways upon the activation of S100A12, and the S100/RAGE interaction accelerates the development of cardiac hypertrophy and diastolic dysfunction in mouse models of CKD (49), further increasing mortality in uremia patients. TNNT1 has been associated with myopathy and even some cancers, but there are no definitive results on the mechanisms affecting uremia, which need to be further investigated. RAB13, which is mainly associated with the trafficking of intracellular material and the functional regulation of organelles, is similar to TNNT1, and the relationship to the role of uremia is unknown. IL7R and CD3D have been found to have a possible relationship with nephropathy, especially diabetic nephropathy, in previous studies, and similarly, RETN (resistin) has been found to play a role in diabetic nephropathy as well as renal insufficiency, but unfortunately, studies have not involved pathogenesis, and basic experiments are also needed for further exploration. Recently, experts have found common pathways and protein expressions in the central nervous system (CNS) and kidney, including glutamate signaling (50), nephrin expression (51), and podocalyxin expression (52), which also serve as the basis of our study. Through these findings, it is understood that brain-derived neurotrophic factor (BDNF), which is primarily produced in the nervous system, is also secreted by the kidneys. To investigate BDNF function *in vivo*, Endlich et al. knocked down BDNF in zebrafish larvae and found that it led to decreased expression of podocin and nephrin, as well as enlarged Bowman’s spaces, glomerular telangiectasia, and podocyte loss. These structural changes were associated with an increased urinary albumin–creatinine ratio. Based on these findings, BDNF has been suggested as a novel potential biomarker of glomerular kidney injury (53). BDNF is associated with sarcopenia (54), insulin resistance (55), depression (56), and inflammation (57). Because all these adverse conditions are also present in CKD patients, and BDNF is expressed in glomeruli and tubules, Trk receptors (TrkB and TrkC) are expressed in proximal and distal tubules, as well as in collecting duct epithelial cells (58). It can be speculated that BDNF may be a potential marker of CKD. Many researchers have investigated the relationship between depression and BDNF in CKD. Sun et al. showed that the uremic toxin indoxyl sulfate is associated with mood disorders and neurodegeneration and has an inhibitory effect on BDNF expression in unilateral nephrectomized mice (59). Similar results showed that *p*-cresol sulfate (PCS) levels were increased and BDNF was decreased in C57/BL/6 mice after unilateral nephrectomy, and these changes were often accompanied by depression-like, anxiety-like, and cognitive impairment behaviors (60). However, studies on depression and BDNF have not been consistent, and Alshogran et al. showed that BDNF concentrations did not correlate with depression scores (61). Overall, BDNF may reflect a promising marker for depression screening in CKD. The investigation of BDNF is mainly in the screening of depression, and whether it is a biomarker of CKD or even uremia still needs to be further explored.

At present, the treatment of uremia is scarce and expensive, and the development of new therapeutic drugs is not easy. Therefore, the use of the DSigDB database to find potential therapeutic agents against uremia-related causative genes provides new insights into the treatment of uremia. Importantly, it not only shortens the time but also significantly reduces the cost of developing drugs. Previous studies have shown that uremic toxins may inhibit Klotho expression by promoting increased DNA methyltransferase expression and DNA hypermethylation (62). At the same time, Klotho, as a renoprotective factor (63, 64), is significantly decreased in uremia patients (65). Decitabine prevents early kidney damage by inhibiting DNA methyltransferases, reducing methylation of DNA, and increasing Klotho expression (66). Of course, when it progresses to end-stage renal disease, dialysis is the main treatment, and different dialysis methods will also cause various injuries to patients (67), which is also the direction to be explored in the future.

## 5 Limitations

Our study has several limitations. Despite including three datasets from the GEO database to mitigate the impact of a single sample, the volume of collected data requires augmentation due to the numerous models we analyzed. Furthermore, while we successfully validated our model’s predictive performance, additional experimental studies are needed to further confirm our biomarkers and mechanisms of action.

## 6 Conclusions

Our research establishes a novel molecular framework for the early diagnosis of uremia, especially in patients diagnosed with MDD. Furthermore, we have conducted extensive model analyses and identified an optimal diagnostic model, which provides valuable insights for more comprehensive and effective diagnostic gene analysis.

## Data Availability

The original contributions presented in the study are included in the article/supplementary material. Further inquiries can be directed to the corresponding author.
